# To Bleed or Not to Bleed

**DOI:** 10.7759/cureus.37591

**Published:** 2023-04-14

**Authors:** Peter A Iskander, Syed Muhammad Hussain Zaidi, Jiayi Zheng, Anthony Iskander, Mark M Aloysius, Vikas Khurana, Simin Nasr

**Affiliations:** 1 Internal Medicine, The Wright Center for Graduate Medical Education, Scranton, USA; 2 Internal Medicine, Xavier University School of Medicine, Oranjestad, ABW; 3 Gastroenterology, The Wright Center for Graduate Medical Education, Scranton, USA; 4 Family Medicine, The Wright Center for Graduate Medical Education, Scranton, USA

**Keywords:** direct oral anticoagulant (doac), doac, anticoagulation, endoscopy, gastrointestinal bleed

## Abstract

The location of gastrointestinal (GI) bleeding can be characterized based on the characteristics of the bowel movements these patients present with. Bright red blood per rectum is usually associated with a lower bleed; if brisk and significant enough, however, upper bleeds can present similarly. Melenic or “tar-colored” bowel movements are more likely to occur from upper bleeds as the color is secondary to digestion of hemoglobin as it passes through the GI tract. At times, there can be a mix of the two which can make a clinical decision for intervention less obvious. To make matters more difficult, some of these patients can be on anticoagulation therapy for a myriad of reasons. Risks versus benefits at these times need to be weighed as holding this therapy may make the patients more prone to clotting while continuation would increase likelihood of bleeding. We present a case of a hypercoagulable patient who was started on rivaroxaban for history of pulmonary embolism; this led to the onset of an acute GI bleed from a duodenal diverticulum requiring endoscopic intervention. Although this can be a known effect of the therapy, the severity of bleed and changes in hemodynamics can warrant very different management strategies.

## Introduction

Bleeding from the gastrointestinal (GI) tract can originate anywhere from the esophagus to the rectum, with a vast range of symptoms and findings. Bright red blood per rectum is usually associated with a fresh distal bleed in the lower tract while darker melenic stools are more associated with digested blood originating higher up [1]. Depending on the intensity and etiology of the bleed, however, these findings can change. Up to 30% of those with large brisk upper GI bleeds may present with hematochezia; the blood passes swiftly through the tract, therefore not having enough time for proper digestion [2]. The indication to resume, initiate, or discontinue anticoagulation in these patients can be a complicated decision. Those who are in a hypercoagulable state or at risk for clotting would ideally require to be on some kind of anticoagulation regime to help prevent them from clotting; initiation of these medications, however, would put them at risk of worsening their bleed [3].

## Case presentation

An 89-year-old male with a past medical history of multiple myeloma and pulmonary embolism (PE) was admitted for generalized weakness. Of note patient did not recall the details of his PE but noted it was years in the past and that he had never followed up with a physician regarding it. He denied remembering being initiated on any anticoagulation for his PE history. For such, he was started on a planned rivaroxaban regimen of 10mg daily. Unfortunately, he developed both hematochezia and melena the following day. At this time the decision was made to discontinue his anticoagulation. On examination, the patient was pale, diaphoretic, and hypotensive. His abdomen was soft and mildly tender without signs of peritonitis. Hemoglobin on presentation was 8.5 g/L (normal: 14 to 18 g/dL). Intravenous (IV) crystalloids and pantoprazole infusion were initiated. Despite being transfused packed red blood cells, platelets, and fresh frozen plasma (FFP), he remained hemodynamically unstable. On evaluation blood pressure was noted to be 92/65 mmHg with a heart rate of 117 beats per minute. Ultimately, the decision was made to transfer to the intensive care unit (ICU) where he was started on norepinephrine drip.

**Figure 1:**
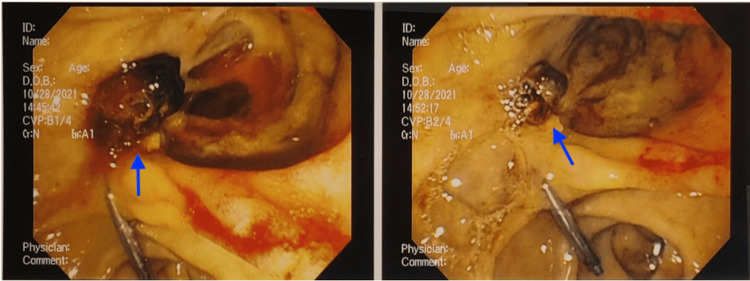
Esophagoduodenoscopy (EGD) photo taken with arrow indicating adherent clot in a duodenal diverticulum before (left) and after removal and clipping (right).

Emergent esophagoduodenoscopy (EGD) was performed, which showed a large clot in a duodenal diverticula with surrounding active bleed. 4 mL of 1:10,000 diluted epinephrine was injected around the site (12, 3, 6, 9 o'clock positions) and the clot was removed. Due to persistence of the bleed, interventional radiology was consulted for which he was taken for sub-selective embolization of the gastro-duodenal and supra-duodenal arteries. Patient was monitored the following day; his hemodynamics were noted to improve and was transferred out of the ICU to the general medical floors. Later that evening he proceeded to have another bloody bowel movement for which repeat EGD was done. Oozing was appreciated around a new clot within the diverticula and 3 mL of diluted 1:10,000 epinephrine was circumferentially injected this time. A clip was placed which helped achieve hemostasis and the clot was then removed via biopsy forceps (Figure 1). Hemodynamics improved after the second EGD. Due to reports of further melena over the next few days, a third EGD was performed; no further evidence of bleeding was noted. He was restarted on his anticoagulation; his hemodynamic remained stable and he was later discharged with instructions for close follow-up.

## Discussion

Treatment modalities for evidence of GI bleed can vary based on intensity and location. Patients who are hemodynamically stable may just warrant observation and supportive care via IV fluids [4]. Although our patient was noted to have an upper GI bleed in the duodenum, most GI bleeds are secondary to diverticula in the lower GI tract; they can account for 20.8 to 41.6% of cases [5]. These cases have become more prominent as of late secondary to increase use of antiplatelets and anticoagulation in the elderly population [6]. Further evaluation can be done via endoscopic approach for which direct visualization of a bleeding vessel can be identified; interventions can then be implicated. For example, cauterization of bleeding vessels can be done, clipping, or, as in our case, epinephrine injections to help with local vasoconstriction [7]. The indication for anticoagulation in patients with evidence of bleeding is a difficult decision to make. Those in hypercoagulable states (i.e. ones at higher risk for developing thromboembolisms) should ideally be on an anticoagulation regimen for prevention of clot formation [8]. This, however, runs the risk of worsening or even precipitating a bleed. Studies have been done regarding the appropriateness of restarting anticoagulation in patients who have experienced some kind of GI bleed. In one such study looking at the resumption of warfarin, it was noted that the recurrence of the bleed (hematemesis, hematochezia, melena, etc) was significantly increased if initiated within seven days; death rate was at its lowest when restarted within 15-90 days [9]. Most re-bleeding complications seem to occur when anticoagulants are resumed within the first week of the initial bleed; results are most favorable when restarted between seven to 30 days with preference after two weeks [9]. Decision for discontinuation of anticoagulation can be determined based on severity and hemodynamic status; those with minor rectal bleeding may continue or delay their next dose, however, hemodynamic compromise would warrant discontinuation [9]. 

For those already on anticoagulation and presenting with bleed, the indications for reversal can be determined based off of the type of anticoagulation regimen the patient is on [10]. Per the American College of Gastroenterology and the Canadian Association of Gastroenterology Clinical Practice Guidelines, those on warfarin should be given prothrombin complex concentrate (PCC) as opposed to vitamin K and FFP. This differs from those on direct oral anticoagulants, in which case PCC would be indicated; they did advise against administration of andexanet alfa [11]. This can be correlated to our patient on rivaroxaban therapy for which clotting factors were repleted via FFP, as opposed to PCC, due to faster availability at the time. 

## Conclusions

As the number of patients being placed on anticoagulation therapy increases, so do the cases of GI bleeds. Careful consideration needs to be made to determine the necessity and contraindications associated. Those with minor bleeds may be able to continue their regimen with close monitoring, supportive care, and close follow-up. Those with more substantial bleeds and hemodynamic compromise, however, would require a risks versus benefits discussion. Determining the necessity of continuation should be addressed while noting the increase risk of bleed versus clotting. Co-morbidities, their thromboembolic risk, and the bleeding intensity should all be considered. Regardless, endoscopic follow-up would likely be warranted to help rule out other associated bleeding pathologies (i.e. ulcers, arteriovenous malformations, etc). As with our case, anticoagulation initiation did result in bleeding. Appropriate fluid resuscitation and EGD intervention were successful in improving hemodynamics. After some close monitoring for further bleed, he was able to resume his anticoagulation and be discharged in stable condition. Despite studies and results, the decision of resumption of anticoagulation should be made on an individual patient basis.
